# Performance of Verbal Fluency as an Endophenotype in Patients with Familial versus Sporadic Schizophrenia and Their Parents

**DOI:** 10.1038/srep32597

**Published:** 2016-09-01

**Authors:** Sugai Liang, Wei Deng, Qiang Wang, Xiaohong Ma, Mingli Li, Matthew R. G. Brown, Xun Hu, Xinmin Li, Andrew J. Greenshaw, Tao Li

**Affiliations:** 1 Department of Psychiatry and Psychiatric Laboratory, State Key Laboratory of Biotherapy, West China Hospital, Sichuan University, Chengdu, Sichuan 610041, China; 2 West China Brain Research Centre, West China Hospital, Sichuan University, Chengdu, Sichuan 610041, China; 3 Department of Psychiatry, University of Alberta, Edmonton, AB T6G2B3, Canada; 4 Huaxi Biobank, West China Hospital, Sichuan University, Chengdu, Sichuan 610041, China

## Abstract

What’s the neurocognitive deficit as an endophenotype to familial schizophrenia? Here, we investigate the neurocognitive endophenotype in first-episode patients with familial schizophrenia (FS) and sporadic schizophrenia (SS), and their parents. 98 FS patients and their 105 parents; 190 SS patients and their 207 parents; 195 controls matched with patients, and 190 controls matched with the patients’ parents, were assessed with the short version of the Wechsler Adult Intelligence Scale-Revised in China (WAIS-RC), the immediate and delayed logical memory tests from the Wechsler Memory Scale-Revised in China (WMS-RC), the Verbal Fluency Test (VFT), the Trail Making Test Parts A and B-Modified (TMA, TMB-M), and the Modified Wisconsin Card Sorting Test (WCST-M). The results showed that with age, gender, and education as covariates, after controlling for false discovery rates, the FS group and their parent group performed worse than the SS group and their parent group on VFT. No significant differences were found for other neurocognitive tests between the FS and SS patient groups, and their respective parent groups. Our findings suggest the patients with familial and sporadic schizophrenia and their respective parent groups may have a different genetic predisposition in relation to a cognitive endophenotype.

Cognitive deficits are core features of schizophrenia and are considered as putative endophenotypes. Cognitive impairment associated with schizophrenia is severe, and widespread^1^,^2^,^3^. Both schizophrenia and cognitive impairment are highly heritable^4^,^5^. As Gottesman and Gould^6^ defined, endophenotypes measure components unseen by the unaided eye along the pathway between disease and distal genotype, which may provide a more reliable index of liability than disorder itself. Previous literature reports that individuals with schizophrenia, compared with normal controls, have deficits in verbal memory, speed of cognitive processing, working memory, and verbal fluency^7^,^8^,^9^.

Cognitive deficits that occur in individuals with schizophrenia may also be observed in non-affected relatives in relation to similar cognitive deficiencies. Increasing familial evidence indicates that cognitive impairment, as an endophenotypic feature, may also be observed in non-unaffected relatives at a higher rate than in the general population^10^,^11^,^12^,^13^. Unaffected relatives of individuals with schizophrenia may also have cognitive impairment in working memory, sustained attention, set shifting, and inhibition^14^,^15^,^16^. Moreover, cognitive deficiencies in unaffected relatives, potentially caused by familial predisposition to schizophrenia are also stable and heritable^13^,^17^,^18^. Cognitive impairment in high risk relatives may predict later emergence of psychopathology and is a possible pathognomonic target for early intervention^19^, although, how family history has an effect on the cognitive impairment of unaffected schizophrenic relatives remains unclear.

Among the various approaches to interpret endophenotype and complex gene–environment interactions, the familial-sporadic distinction is a division based on genetic risk related to family history status. Murray, *et al*.^20^ defined that familial schizophrenia (FS) is associated with a positive family history of psychotic disorders, whereas sporadic schizophrenia (SS) is not. Roy, *et al*.^21^ reported that FS was mainly influenced by genetic factors whereas SS with low genetic risk was a more environment-related subtype. Some previous studies suggest that FS is the more serious subtype with more minor physical anomalies, more cognitive impairment, and severe structural brain abnormalities compared to SS^22^,^23^,^24^,^25^. However, Birkett, *et al*.^26^ did not find any difference in neuropsychological functioning between schizophrenic patients with and without a family history. Similarly, a recent resting–awake electroencephalographic study also reported that the two patient subgroups did not differ on dense array spontaneous gamma oscillatory activity^27^.

Previous studies have also shown that more structural abnormalities may be found in schizophrenic individuals without a family history^28^,^29^. Griffiths, *et al*.^30^ reported that sporadic schizophrenic individuals had more minor physical anomalies compared to familial cases. Additionally, our previous neuroimaging data revealed that brain white matter deficits were more severe in a non-familial than in a familial schizophrenia subgroup^31^. These inconsistent findings required to clarify further the sporadic/familial distinction of schizophrenia in relation to cognitive function.

The purpose of this investigation is, firstly, to understand how familial and sporadic schizophrenia patients differ with respect to neurocognitive functioning, which may help to explain the heterogeneous neuropsychological profile of the illness. By better characterizing the neurocognitive profiles of these groups, we may add to knowledge concerning the etiology of potential neuropsychological differences between FS and SS cases. Secondly, the current study examined the same neurocognitive features in unaffected parents of FS and SS cases, to test the hypothesis that a cognitive impairment is associated with their genetic loading for schizophrenia, and to further develop the concept of a neurocognitive endophenotype for this complex disorder.

## Results

### Demographic and clinical characteristics

The FS and SS patient groups and their respective matched control groups did not differ in age, gender, or years of education (Table 1), nor did the FS and SS parent groups, and matched control groups (Table 2). The FS and SS patient groups did not differ significantly with respect to PANSS scores, or to duration of untreated psychosis.

### Neurocognitive function in FS and SS patients

**Figure 1:**
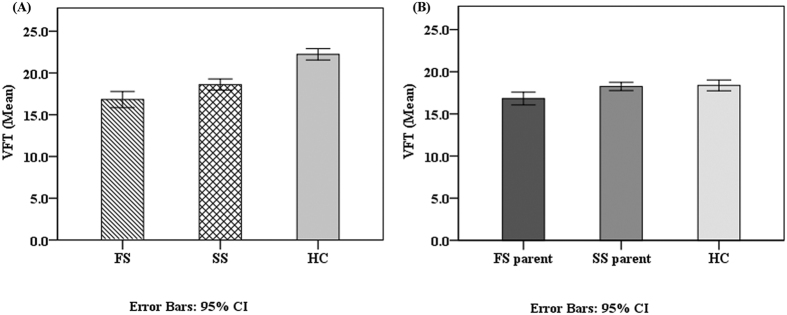
The verbal fluency scores comparisons in patients and their respective parent groups. Cognitive function as measured by the verbal fluency test (VFT) in (**A**) Familial schizophrenic patients (FS), sporadic schizophrenic patients (SS) and healthy controls (HC); (**B**) FS parents, SS parents and HC.

ANOVA revealed significant differences across the patient and healthy control groups for all cognitive tests (Table 3). In comparison with their respective matched controls, both FS and SS individuals exhibited significantly worse performance on all cognitive tests. Compared to the SS group, the FS group performed significantly worse on VFT (p = 0.007, d = 0.38) (Fig. 1). There were no significant differences between the FS and SS groups on other neurocognitive tests.

ANCOVA was performed to rule out the effects of age, gender and educational level. Significant effects across the patient and healthy control groups were found for all cognitive tests. The main effect of VFT among three groups was significant, F (2, 477) = 50.37, p < 0.001. Compared to the SS group, the FS group performed significantly worse on VFT (p = 0.003). No significant differences were found between FS and SS groups on other neurocognitive tests.

### Neurocognitive function in FS parent and SS parent groups

**Figure 2:**
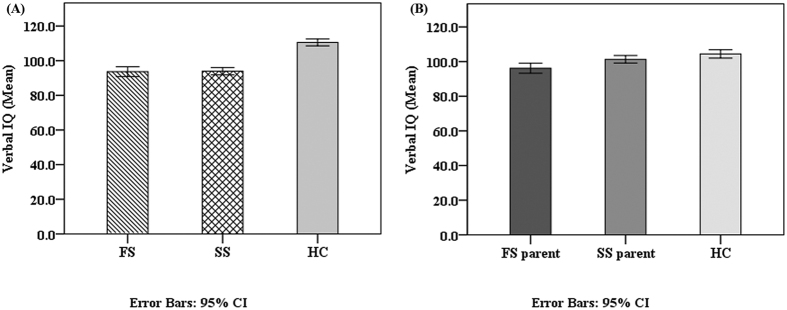
The verbal IQ scores comparisons in patients and their respective parent groups. Cognitive function as measured by the verbal intelligence quotient (Verbal IQ) in (**A**) Familial schizophrenic patients (FS), sporadic schizophrenic patients (SS) and healthy controls (HC); (**B**) FS parents, SS parents and HC.

Significant effects across the schizophrenic parent and healthy control groups were found for all neurocognitive tests except TMA and performance IQ in ANOVA (Table 4). Compared to the healthy control group, the FS parent group exhibited worse performance on all cognitive tests except performance IQ and TMA. Compared to the healthy control group, the SS parent group had worse performance on immediate logical memory (p = 0.001, d = 0.69), delayed logical memory (p = 0.001, d = 0.61), TMB-M (p = 0.03, d = −0.29), WCST-perseverative error (p < 0.001, d = −0.76) and WCST-achieved categories (p < 0.001, d = 0.47). No significant differences were found between **S**S parent and healthy control groups on verbal IQ, performance IQ, IQ, VFT, and TMA. Compared to the SS parent group, the FS parent group performed significantly worse on VFT (p = 0.01, d = 0.39) (Fig. 1), and Verbal IQ (p = 0.02, d = 0.34) (Fig. 2). No significant differences were found between FS parent and SS parent groups on other neurocognitive tests.

Controlling for age, gender and education level, significant effects among schizophrenic parent and healthy control groups were found for all neurocognitive tests except TMA. The main effect of performance IQ among the three groups was significant, F (2, 496) = 3.78, p = 0.02. Compared to the healthy control group, the FS parent group was worse on all cognitive tests except performance IQ and TMA. Performance of the SS parent group was worse than the healthy control group on all neurocognitive tests except VFT and TMA. Partialing out the effect of covariates with FDR correction, the performance of the FS parent group was worse than that of the SS parent group on VFT (p = 0.026). No significant differences were found between the FS parent and SS parent groups on verbal IQ, performance IQ, and other neurocognitive tests after controlling for age, gender, and education level.

### Partial correlation analysis

**Figure 3:**
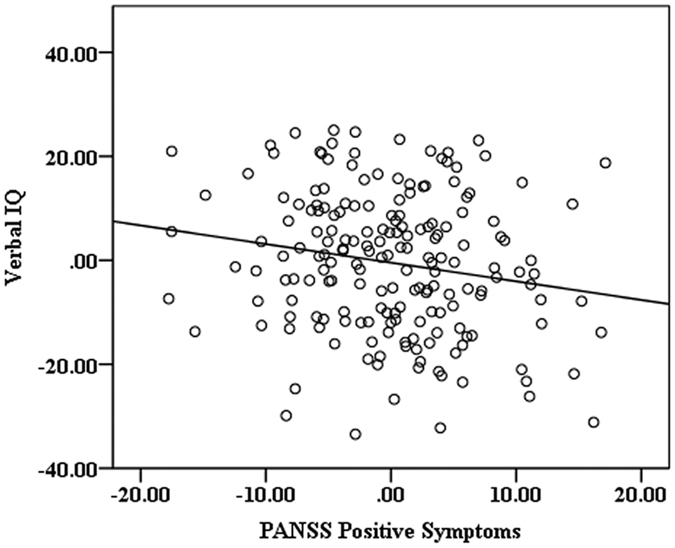
Partial correlation for positive symptoms and verbal IQ in patient groups. Age, gender and education level were included as covariates. r = −0.17, p = 0.03. The x axis represents residuals of PANSS positive symptoms scores, the y axis represents residuals of verbal IQ.

The partial correlation analysis revealed that verbal IQ was significantly correlated with PANSS positive symptoms (r = −0.17, p = 0.03) in patient groups (Fig. 3). There were no other significant correlations between clinical symptoms and other cognitive tests scores. No significant correlation was found in duration of untreated psychosis and neurocognitive scores in the two patient groups after controlling for age, gender and education level. No significant correlations were observed for clinical symptoms and neurocognitive tests in either the FS group or the SS group after controlling for the covariates.

## Discussion

This study is based on a large research sample and focused on the neurocognitive impairment of individuals with first-episode schizophrenia and their parents. The current study revealed that compared to SS individuals and their parents, FS individuals and their parents had a greater impairment of verbal fluency, the effect was observed with or without ANCOVA. Notably, the current findings indicated that the consistency of verbal fluency deficiency in schizophrenic individuals and their unaffected parents was highly related to the family history. The results of the present study provide further evidence concerning the link between family history and definition of a cognitive endophenotype in schizophrenia.

Intriguingly, the current findings indicate that FS individuals had worse performance than SS individuals on verbal fluency. This cognitive deficit pattern was also observed with respect to their unaffected parents. As we know, verbal fluency tests are widely used as a tool to assess verbal ability and executive control^32^. Results from a prior longitudinal study indicate that semantic verbal fluency could be a promising potential endophenotype in schizophrenia^1^. Dysfunction of semantic fluency is one of the core features of schizophrenia, and is independent of language system or cultural backgrounds^33^. Recent research also demonstrates that semantic fluency function could reveal potential endophenotypes for the early diagnosis of schizophrenia in the Han Chinese population^34^. Children who later developed schizophrenia, and their siblings, showed similar cognitive deficits and, compared to siblings of unaffected individuals, the probands exhibited more severe deficits in semantic fluency function^35^,^36^. Siblings of schizophrenic individuals may exhibit significantly less word output in the verbal fluency test, which probably indicated semantic verbal fluency deficit as a familial trait marker in schizophrenia^37^. Semantic verbal fluency differences may be associated with a large effect size between relatives of schizophrenic cases and controls^38^.

In present study, we studied parents rather than siblings^37^ of patients provided additional evidence to support the hypothesis that verbal fluency may be an endophenotype in familial schizophrenia. There was a significant difference in verbal fluency impairment not only in familial schizophrenic individuals but in their unaffected parents. This factor leads us to consider that verbal fluency impairment in schizophrenia may not only be specific to the disorder itself, but also could be a genetic endophenotype. To elucidate further the mechanisms underlying verbal fluency performance relationships between patients and their parents, future research should be combined with neuroimaging and/or genetic studies.

In accord with the results of previous studies, this study investigated the neurocognitive functions of individuals with FS and SS, and identified impairment in both groups^1^,^2^. In the current study, there were no statistically significant differences in verbal, performance or full IQ scores between familial and sporadic schizophrenic individuals. In addition, verbal IQ was related to positive symptoms in schizophrenia groups. Previous research revealed that disorganized symptoms were correlated with lower verbal IQ values^39^, and improved verbal concept formation may be associated with reduction in positive symptoms in schizophrenic patients treated with clozapine^40^. Schizophrenia with positive symptomatology particularly prone to misattribute their distorted voice to someone else, which may reflect impaired verbal self-monitoring^41^,^42^,^43^. The predisposition to experience auditory verbal hallucinations is associated with aberrant language performance, which might be related to difficulties in the inhibition of irrelevant verbal information^44^. However, the current study didn’t find the significant correlation between neurocognition and negative symptoms.

Notably, there is also evidence that FS parents tended to have more neurocognitive deficits than SS parents in relation to general intelligence and executive function. Although FS and SS parent groups exhibited no clinically significant differences, scores of FS parents were worse than SS parents on verbal IQ. When we partial out the effect of covariates, no significant difference was found between the two parent groups on IQ. Similarly, comparisons between FS relatives and SS relatives showed that the former group had significantly worse scores for estimated intelligence, logical memories, immediate visual reproductions and the WCST^45^,^46^. But in the study of Erol, *et al*.^25^, no significant differences were found between SS parents and their controls on any of the tests except for the Stroop color score, however, FS parents performed significantly worse than SS parents on the VFT, the TMT, the WCST and the Stroop Test. Erol, *et al*.^25^ demonstrated that executive functions were impaired only in parents with a positive family history of schizophrenia. The current study not only provided further evidence the conclusion on subclinical subjects with a relative large sample size, but reduced the confounding factors such as medication and chronic duration of illness significantly as all patients in present study were first-episode schizophrenia and the majority of them (275 out of 288, 95%) were treatment-naïve at the time of cognitive function assessment.

According to the multifactorial/threshold model, various risk factors accumulate up to a threshold level for clinical manifestation of schizophrenia, which could explain the gene-environment contribution to the disorder^47^. When individuals have high genetic loading, even minor environmental insults may be sufficient to trigger the onset of illness. Because of their less severe irreversible brain damage, these individuals with minor brain insults might have a better clinical outcome^26^,^37^. By contrast, sporadic individuals with a form of schizophrenia associated with a lower genetic loading could have undergone more severe environmental damage leading to cerebral insult. Confirmed *de novo* number variation (CNV) mutations were collectively ~8 times more frequently in SS (but not FS) cases than in unaffected controls^48^. There is a strong association of *de novo* CNV with sporadic cases. The increase in minor physical abnormalities in SS individuals, especially males, which probably supports the notion that abnormality of prenatal development is particularly implicated in SS^30^. In our previous study, the SS had more severe brain white matter abnormities than FS subgroup in the left temporal lobe and right corpus callosum^31^. However, the left inferior/middle frontal gyrus has consistently been associated with semantic verbal fluency in functional neuroimaging research^49^,^50^,^51^. The impaired prefrontal function in schizophrenia is also related to the verbal fluency^52^. Overall, because of the complex nature of the disease, and inconsistencies of the literature, it is difficult to categorical statement that FS individuals have more neurocognitive impairment than SS individuals.

There are, however, some limitations that must be considered when interpreting our findings. The first limitation is the possibility of environmental factors acting on “vulnerability to schizophrenia”. Secondly, the current study didn’t include the siblings/offspring of schizophrenic individuals. Our previous study provided evidence for a hierarchy in cognitive performance deficits depending on the degree of relatedness to probands^53^. This hierarchy of cognitive impairment should be considered in future studies. Thirdly, the presence of positive and negative symptoms in the parent groups were not assessed: the inclusion of individual symptoms in the analyses might have helped further clarify the specificity of association between neurocognition and symptoms^54^. Fourthly, although there was no significant high intra-correlation of both parents from the minority of patients in each parent group, even results without the thirteen SS parents didn’t show significant changes, one parent or both parents from the patient was still a confounding factor in the current study.

In conclusion, the current study provides more evidence for the hypothesis that impaired cognitive functions could represent an endophenotypic marker of vulnerability to schizophrenia. The verbal fluency deficient could represent an endophenotype in familial schizophrenia. Our results also revealed that parents with a positive family history performed significantly worse than those without such a history. Our findings suggest that the amount of neuropsychological impairment in relatives of schizophrenic individuals may increase with their genetic loading for schizophrenia, especially in the familial schizophrenia subtype.

## Methods

### Participants

The current study recruited 288 first-episode patients diagnosed with schizophrenia (98 with FS and 190 with SS) recruited from in-patient and out-patient psychiatric units in the West China Hospital, Sichuan University, along with 105 unaffected parents of FS patients, and 207 unaffected parents of SS patients that met the study criteria. This study also included 195 healthy controls that matched the patients and 190 healthy controls that matched the patients’ parents with respect to age, gender and education level, resulting in a total population of 985 subjects.

Inclusion criteria were as follows. All participants are right handed Han Chinese between the ages of 16 and 65 years old. Patient participants had a diagnosis of schizophrenia or schizophreniform disorder according to DSM-IV^55^, assigned on the basis of the interview and medical records. In present study, we defined the patients with first episode schizophrenia if they had the first presentation to psychiatric services as a result of psychotic symptoms according to the Johnstone, *et al*.^56^. In order to clarify diagnosis in patients presenting with a first episode of psychosis, we also had the prospective longitudinal observation to those patients who had the short duration of illness (e.g. less than 6 months) for at least 6 months.

Exclusion criteria included any history of neurological disorders, head traumas, intellectual disability or medical conditions that might alter cognitive functioning. Those who had current or recent substance use disorder (within the past month), psychoses secondary to medical illness, organic brain syndrome, or learning disability were also excluded. In addition, subjects were excluded if they had significant medical illness or if they were judged clinically to have a high risk of suicidal behavior. Healthy control subjects reporting mental disorders in their first-degree relative were also excluded. All participants were interviewed using the Structured Clinical Interview for DSM-IV: SCID-NP for controls^57^ and SCID-P for patients^58^. All assessments were conducted within the first three days of medication.

In present study, the majority of patients (275 out of 288) were treatment-naïve at the time of cognitive function assessment, but thirteen out of 288 patients had been minimally treated with antipsychotics such as risperidone or olanzapine at low dosage (ranging from 25 to 75 mg of chlorpromazine daily dose equivalents) for a brief duration of less than 3 days. The medicated patients were also evaluated with the Treatment Emergent Symptom Scale (TESS).

Patients were considered to have a positive family history, if at least one first or second degree relative had a severe non-affective psychosis (schizophrenia, schizoaffective, psychosis NOS and completed suicide). If not, patients were defined as family history negative or sporadic cases. Family psychiatric history was obtained by interviewing the patients and both parents. During the interview, we also asked whether their relatives (within first and second generations) had mood disorders, substance abuse history or some psychotic symptoms before the suicide completion or suicide attempt. If the relatives didn’t have mood disorders or substance abuse history, but some psychotic symptoms before suicide, they were included to the current study as familial schizophrenia. Other first-degree relatives (i.e. siblings and offspring) were interviewed, where possible, to provide additional information on family history during the clinical interview. Medical records of first-degree relatives were used to confirm positive family history.

In the FS group, thirteen out of 98 had both parents; in the SS group, nineteen out of 207 had both parents. In the FS group, six patients (1 female/5 male, average age 34.00 ± 4.13 years old) didn’t have parent in the FS parent group. In the SS group, fifteen patients (9 female/6 male, average age 32.00 ± 2.44 years old) didn’t have parent in the SS parent group. Reasons were followings: some parents were more than 60 years old with low education level, they could not complete the neurocognitive tests; some directly refused to take the tests for themselves. In the SS parent group, thirteen parents (8 female/5 male, average age 35.00 ± 0.48 years old) did not have children in the SS group. These thirteen parents were from the individuals with early onset schizophrenia less than 16 years old.

Healthy controls were recruited by advertisements in local communities. Written informed consent was obtained from all participants after the procedure had been fully explained. Ethical approval was obtained from the local research ethics committee according to the Declaration of Helsinki. This study was approved by the Institutional Research Ethics Broad (IRB) of West China Hospital, Sichuan University.

### Assessment methods

#### Clinical assessment

Symptom severity was evaluated using the Positive and Negative Syndrome Scale (PANSS) which is commonly used to assess positive and negative symptoms, and general psychopathological symptoms in schizophrenia^59^. All items were rated from 1 (absent) to 7 (extreme) according to standardized instructions.

#### Neurocognitive assessments

All neuropsychological tests reported here were also included in our previous studies, to analyze neurocognitive deficits in first-episode schizophrenic patients and their first-degree relatives^11^,^60^.

Level of intelligence was evaluated at the first assessment of both patients and healthy controls using the short version of the seven-subtest (information, arithmetic, digital symbol, digital span test, block design, picture completion, and similarities) Wechsler Adult Intelligence Scale – Revised in China (WAIS-RC)^61^. For the short form^62^, the Verbal sum of scaled scores was obtained as follows: 2 (Information + Similarities) + Arithmetic + Digit Span; Performance sum was calculated by 2 (Picture Completion + Block Design) + Digit Symbol. Full Scale IQ estimates were based on the Ward Verbal + Performance sums. The estimated sums of scaled scores derived from these formulae were then converted to IQ scores using the standard procedure and age-corrected conversion tables in the WAIS-RC manual.

Memory was evaluated using the immediate and delayed logical memory tests from the Wechsler Memory Scale – Revised in China (WMS-RC)^63^. Lower raw scores represent poorer neuropsychological performance.

Verbal fluency (VFT)^37^ was assessed in letter and category fluency tasks, and performance on these tasks was related to indicators of vocabulary size, lexical access speed, updating, and inhibition ability. This study utilized category naming, the most commonly used VFT, where subjects are asked to generate example words for a given category (e.g. animal or fruit) in a specified time limit: we evaluated the number of valid words pronounced in 60 seconds.

Processing speed during attention and working memory were measured with the Trail Making Test (TMT), parts A (TMT-A) and B-Modified (TMT-B-M). This is a test of complex visual scanning with a motor component which can evaluate the flexibility in shifting the course of an ongoing activity. The purpose of the TMT is to measure visual scanning, conceptual flexibility, and motor speed. Final scores are measured as the time taken to complete each part of the task. TMT-A requires an individual to draw lines sequentially connecting 25 encircled numbers distributed on a sheet of paper. Task requirements are similar for TMT-B-M except the person must alternate between numbers and Chinese letters. We used test duration time for this test. Score was the total time required to complete the task, that high scores meant worse performance^64^,^65^.

The Modified Wisconsin Card Sorting Test (WCST-M)^66^ was adopted to assess the executive function of participants, the number of perseverative errors and categories achieved were recorded. Each participant’s first sorting choice becomes the correct feature, and once a criterion of six consecutive correct sorts is achieved, the subject is told that the rules have changed, and cards must be sorted according to a new feature. The sorting rules could be color, shape, or number. After all three features have been used as sorting criteria; subjects must cycle through them once again in the same order as they did before. Each time the feature is changed, the next must be discovered by trial and error. Scores were perseverative errors and total number of categories achieved before completing a maximum of 48 cards. The number of perseverative errors was calculated to reflect cognitive flexibility, whereas the number of categories completed was recorded to measure attention allocation and planning^67^.

### Data analysis

Descriptive statistics were computed for basic demographic and clinical variables. Gender distribution was analyzed using Chi-square; continuous variables (age, years of education, and cognitive tests scores) were compared with one-way analysis of variance (ANOVA). Post-hoc multiple comparisons were conducted with Tukey’s HSD tests. Effect sizes were calculated with Cohen’s d to examine how well these neuropsychological tests could separate two groups of individuals^68^. According to Cohen’s convention, a small effect size corresponds to 0.20, a medium effect size corresponds to 0.50, and a large effect size corresponds to greater than 0.80. Patients’ duration of untreated psychosis and PANSS scores were compared with the independent-samples t test. Analysis of covariance (ANCOVA) was conducted to assess differences in neuropsychological tests, with age, gender and years of education as covariates. The False discovery rate (FDR) is a common way of conceptualizing the rate of type I errors when conducting multiple comparisons. To control the FDR between groups after ANCOVA, the method of Benjamini & Hochberg (BH) (1995) was used employing an in-house program using R language. Partial correlation analyses were conducted between duration of untreated psychosis and cognitive scores controlling for age, gender, and education level. The relationship between the neurocognitive deficits and clinical profiles was analyzed with partial correlation analysis with age, gender, education level, and duration of untreated psychosis of patients as covariates. All tests were two tailed with a significance level of p < 0.05. Unless otherwise stated, data were analyzed using the Statistical Packages for Social Sciences (SPSS) version 17.0 for Windows.

## Additional Information

**How to cite this article**: Liang, S. *et al*. Performance of Verbal Fluency as an Endophenotype in Patients with Familial versus Sporadic Schizophrenia and Their Parents. *Sci. Rep.* **6**, 32597; doi: 10.1038/srep32597 (2016).

## Figures and Tables

**Table 1:** Demographic and clinical characteristics of patient and control groups.

	FS (n = 98)	SS (n = 190)	Control (n = 195)	Statistic
Age, mean (SD), years	23.29 (6.79)	22.61 (6.15)	23.10 (5.45)	F = 0.51,p = 0.60
Gender, M/F	43/55	96/94	100/95	χ^2^ = 1.57,p = 0.46
Education, mean (SD), years	11.80 (2.66)	11.73 (2.90)	12.18 (2.91)	F = 1.34,p = 0.26
Duration, mean (SD), months	19.83 (28.81)	15.40 (22.44)		t = 1.28,p = 0.20
PANSS, mean (SD)				
Total profile symptoms	87.64 (20.83)	85.98 (21.57)		t = 0.49,p = 0.63
Positive symptoms	25.45 (7.36)	23.67 (6.44)		t = 1.67,p = 0.10
Negative symptoms	18.05 (7.11)	18.82 (7.06)		t = −0.69,p = 0.49
General psychopathology	44.14 (12.71)	43.49 (13.26)		t = 0.31,p = 0.76

FS, familial schizophrenia; SS, sporadic schizophrenia; M, male; F, female; PANSS, Positive and Negative Syndrome Scale; SD, standard deviation; *p < 0.05, **p < 0.005.

**Table 2:** Demographic characteristics of parent and control groups.

	FS parent (n = 105)	SS parent (n = 207)	Control (n = 190)	Statistic
Age, mean (SD), years	45.71 (6.27)	46.17 (6.37)	44.98 (7.24)	F = 1.58,p = 0.21
Gender, M/F	45/60	101/106	85/105	χ^2^ = 1.19,p = 0.55
Education, mean (SD), years	8.55 (3.11)	9.19 (2.60)	9.09 (3.04)	F = 1.81,p = 0.17

FS parent, familial schizophrenia parent; SS parent, sporadic schizophrenia parent; M, male; F, female; SD, standard deviation; *p < 0.05, **p < 0.005.

**Table 3:** Comparison of neurocognitive test scores between patient and control groups.

	FS	SS	Control	ANOVA	Tukey’s HSD test
WAIS-RC					
Verbal IQ	93.67 (13.97)	93.95 (14.75)	110.52 (13.93)	F = 79.45**	FS-control p < 0.001**
					SS-control p < 0.001**
					FS-SS p = 0.99
Performance IQ	79.19 (16.08)	76.05 (18.53)	100.82 (15.45)	F = 116.10**	FS-control p < 0.001**
					SS-control p < 0.001**
					FS-SS p = 0.29
IQ	86.69 (13.84)	85.46 (15.35)	107.10 (13.89)	F = 125.27**	FS-control p < 0.001**
					SS-control p < 0.001**
					FS-SS p = 0.77
WMS-RC					
Immediate logical memory	6.58 (3.74)	6.68 (3.52)	13.22 (3.50)	F = 198.43**	FS-control p < 0.001**
					SS-control p < 0.001**
					FS-SS p = 0.97
Delayed logical memory	4.82 (3.54)	4.24 (2.94)	11.08 (3.95)	F = 218.34**	FS-control p < 0.001**
					SS-control p < 0.001**
					FS-SS p = 0.91
VFT, valid words	16.82 (4.83)	18.61 (4.66)	22.23 (4.83)	F = 50.44**	FS-control p < 0.001**
					FS-control p < 0.001**
					FS-SS p = 0.007*^#^
Trail Test					
TMA: time to completion	55.78 (18.23)	56.30 (20.29)	38.55 (13.32)	F = 59.67**	FS-control p < 0.003**
					SS-control p < 0.001**
					FS-SS p = 0.97
TMB-M: time to completion	84.37 (27.52)	88.77 (33.80)	53.14 (14.16)	F = 100.01**	FS-control p < 0.001**
					SS-control p < 0.001**
					FS-SS p = 0.37
WCST-M					
Perseverative errors	10.00 (8.99)	9.46 (7.96)	2.89 (2.36)	F = 75.60**	FS-control p < 0.001**
					SS-control p < 0.001**
					FS-SS p = 0.79
Categories	3.54 (2.07)	3.65 (2.00)	5.57 (0.81)	F = 84.82**	FS-control p < 0.001**
					SS-control p < 0.001**
					FS-SS p = 0.86

FS, familial schizophrenia; SS, sporadic schizophrenia; Mean (Standard Deviation, SD); *p < 0.05, **p < 0.005.

**Table 4:** Comparison of neurocognitive test scores between parent and control groups.

	FS parent	SS parent	Control	ANOVA	Tukey’s HSD test
WIS-RC					
Verbal IQ	96.15 (14.90)	101.33 (15.60)	104.42 (16.74)	F = 9.10**	FR-control p < 0.001**
					SR-control p = 0.13
					FR-SR p = 0.02*
Performance IQ	90.82 (13.47)	92.09 (14.25)	93.88 (14.26)	F = 1.74	FR-control p = 0.42
					SR-control p = 0.18
					FR-SR p = 0.73
IQ	93.18 (13.71)	96.89 (14.47)	99.69 (14.78)	F = 6.94**	FR-control p < 0.001**
					SR-control p = 0.13
					FR-SR p = 0.08
WMS-RC					
Immediate logical memory	6.99 (3.35)	7.60 (3.52)	9.65 (3.48)	F = 25.89**	FR-control p < 0.001**
					SR-control p < 0.001**
					FR-SR p = 0.30
Delayed logical memory	4.73 (3.57)	5.22 (3.38)	7.32 (3.53)	F = 25.60**	FR-control p < 0.001**
					SR-control p < 0.001**
					FR-SR p = 0.47
VFT, valid words	16.82 (3.90)	18.25 (3.60)	18.38 (4.51)	F = 5.73**	FR-control p = 0.004**
					SR-control p = 0.95
					FR-SR p = 0.009**
Trail Test					
TMA: time to completion	53.06 (14.97)	52.85 (14.13)	51.48 (13.14)	F = 0.64	FR-control p = 0.62
					SR-control p = 0.59
					FR-SR p = 0.99
TMB-M: time to completion	86.89 (34.24)	82.83 (23.74)	76.43 (19.63)	F = 6.65**	FR-control p = 0.002**
					SR-control p = 0.03*
					FR-SR p = 0.36
WCST -M					
Perseverative errors	9.57 (7.18)	8.21 (5.91)	4.58 (3.14)	F = 36.32**	FR-control p < 0.001**
					SR-control p < 0.001**
					FR-SR p = 0.09
Categories	3.70 (1.82)	4.11 (1.68)	4.81 (1.22)	F = 19.60**	FR-control p < 0.001**
					SR-control p < 0.001**
					FR-SR p = 0.07

FS parent (FR), familial schizophrenia parent; SS parent (SR), sporadic schizophrenia parent; Mean (Standard Deviation, SD); *p < 0.05, **p < 0.005.
